# Xiaochaihutang Extract Ameliorates Metabolic Dysfunction-Associated Steatohepatitis in Mice and Modulates the Gut–Liver Axis

**DOI:** 10.3390/antiox15101268

**Published:** 2026-10-01

**Authors:** Jiajia Yu, Zhiyuan Liu, Yanping Li, Zhen Liu, Cen Xiang, Yuou Teng

**Affiliations:** School of Bioengineering, Tianjin University of Science and Technology, Number 9, 13Th Street, Tianjin Economic-Technology Development Area, Tianjin 300457, China; 15122170121@163.com (J.Y.); zhiyuanliu2023@163.com (Z.L.); 15295862096@163.com (Y.L.); liuzhen5957@tust.edu.cn (Z.L.)

**Keywords:** Xiaochaihutang, MASH, network pharmacology, AMPK/ACC

## Abstract

Metabolic dysfunction-associated steatohepatitis (MASH) is a progressive liver disease characterized by lipid metabolism dysregulation, insulin resistance, inflammation, and potential progression to severe liver conditions. Xiaochaihutang (XCHT), a traditional Chinese medicine, has been explored for its therapeutic potential. This study aimed to systematically investigate the role and mechanisms of XCHT in the treatment of MASH. Network pharmacology and ultra-high-performance liquid chromatography coupled with quadrupole time-of-flight mass spectrometry (UHPLC-QTOF-MS) were used to identify active components and pathways. In vitro models using free fatty acids (FFAs) and an in vivo methionine-choline-deficient (MCD) diet-induced MASH mouse model were employed to evaluate hepatoprotective effects, metabolic improvements, and gut microbiota modulation. XCHT reduced FFA-induced injury, decreased aspartate aminotransferase (AST)/alanine aminotransferase (ALT) levels, inhibited lipid accumulation (72% reduction in triglyceride, TG), and alleviated oxidative stress (67% decrease in oxygen species, ROS; 92% restoration of glutathione, GSH). It also modulated gut microbiota, increasing beneficial bacteria, and activated the AMP-activated protein kinase (AMPK)/acetyl-CoA carboxylase (ACC) pathway (2.2-fold increase in phosphorylated AMPK, p-AMPK). Quercetin, kaempferol, β-sitosterol, and baicalin were predicted as key active components. XCHT ameliorates MASH through multi-target regulation of the AMPK pathway and gut–liver axis, supporting its potential as a complementary treatment for metabolic liver diseases.

## 1. Introduction

Metabolic dysfunction-associated steatohepatitis (MASH) is a progressive liver disease with a high global prevalence. Its pathological nature is not limited to lipid metabolism disorders and insulin resistance; rather, a core driving factor is severe disruption of hepatic redox homeostasis [1]. This redox imbalance directly induces hepatocyte damage, triggers an inflammatory cascade, and accelerates fibrosis, constituting a key pathological mechanism underlying the malignant progression of MASH [2]. MASH is closely linked to metabolic disturbances, including obesity, type 2 diabetes, and insulin resistance, and is not associated with alcohol consumption. Furthermore, it has the potential to significantly impair liver function. If untreated, MASH can progress to more severe stages, including liver fibrosis, cirrhosis, and hepatocellular carcinoma. Epidemiological studies suggest that approximately 5.27% of the global adult population is affected by MASH, with significantly higher rates observed in individuals with obesity and diabetes [3]. This makes the development of therapeutic strategies based on redox regulation an urgent clinical priority.

Natural products, due to their abundance of multi-target active molecules and excellent antioxidant properties, offer unique advantages in the treatment of metabolic diseases associated with oxidative stress. Xiaochaihutang (XCHT) is a traditional Chinese herbal formula that includes seven herbs: Chai Hu (*Bupleurum falcatum* Linne), Huang Qin (*Scutellaria baicalensis* Georgi), Ban Xia (*Pinellia ternata* Breitenbach), Ren Shen (*Panax ginseng* Carl Anton von Meyer), Sheng Jiang (*Zingiber officinale* Roscoe), Da Zao (*Zizyphus jujuba Miller* var. *inermis* Rehder), and Gan Cao (*Glycyrrhiza uralensis* Fisher) [4]. XCHT is known to strengthen the spleen and liver, regulate qi and reduce fever, and is widely used clinically for the treatment of chronic hepatitis B and other similarly symptomatic disorders [5]. XCHT is believed to have the potential to reduce hepatic inflammation and improve liver function, and a number of its herbal constituents are known to possess hepatoprotective, anti-inflammatory, and antioxidant properties, which are effective in mitigating hepatocellular damage and preventing the further progression of liver disease [6]. Among them, Chai Hu and Huang Qin have been shown to be key components in regulating lipogenesis and reducing liver inflammation [7]. In addition, recent studies have shown that XCHT alleviates CCl_4_-induced oxidative stress and inhibits hepatic stellate cell (HSC) activation by regulating nuclear factor erythroid 2-related factor 2 (Nrf2) to alleviate CCl4-induced oxidative stress and inhibit hepatic fibrosis progression in rats, thus effectively inhibiting the process of liver fibrosis [8].

XCHT contains various natural compounds with well-established redox activity, including quercetin [9], kaempferol [10], and β-sitosterol [11]. Its potential antioxidant and metabolic regulatory functions suggest that this compound may improve MASH by reshaping the hepatic redox microenvironment, activating the AMPK energy metabolism pathway, and modulating gut–liver axis interactions. However, the specific therapeutic efficacy and mechanism of XCHT in MASH are not fully understood.

The goal of this study is to use network pharmacology and molecular docking methods to systematically identify the bioactive compounds, potential molecular targets, and therapeutic mechanisms of XCHT in the treatment of MASH. By integrating these advanced techniques, the study aims to provide a deeper understanding of the pharmacological actions of XCHT, offering a promising therapeutic option for MASH management and contributing to the broader integration of traditional and modern medical approaches.

## 2. Materials and Methods

### 2.1. Network Pharmacology Analysis of XCHT Bioactive Compounds

Based on the criteria of oral bioavailability (OB) ≥ 30% and drug-likeness (DL) ≥ 0.18, the chemical components of XCHT were retrieved from the Traditional Chinese Medicine Systematic Pharmacology Database and Analysis Platform (TCMSP, https://old.tcmsp-e.com/tcmsp.php (accessed on 10 October 2024)) and were analyzed and identified. Target information was standardized using the UniProt database (https://www.uniprot.org/ (accessed on 10 October 2024)), and a “TCM–component–target” network was constructed using Cytoscape 3.9.1 (Cytoscape Consortium, San Diego, CA, USA) after duplicate removal for visualization and analysis. Targets related to MASH were retrieved from GeneCards (https://www.genecards.org/ (accessed on 10 October 2024)), OMIM (https://www.omim.org/ (accessed on 10 October 2024)), and DrugBank (https://go.drugbank.com/ (accessed on 10 October 2024)). These targets were intersected with XCHT targets to identify potential therapeutic targets using Venny (http://www.bioinformatics.com.cn/static/others/jvenn/example.html (accessed on 10 October 2024)). A PPI network (confidence > 0.700) was constructed using the STRING database (https://cn.string-db.org/ (accessed on 10 October 2024)), and key targets were visualized and screened using Cytoscape. GO and KEGG enrichment analyses were performed using the DAVID database (https://davidbioinformatics.nih.gov/ (accessed on 10 October 2024)), and the top 15 GO entries (biological process, molecular function, and cellular component) and the top 20 KEGG pathways were identified to reveal the potential mechanism of XCHT in MASH.

### 2.2. Preparation and Analysis of XCHT

XCHT was chopped and mixed according to the standard prescription (Chinese Pharmacopeia Commission. Pharmacopeia of the People’s Republic of China, 2020 Edition, Part I. Beijing: China Medical Science Press; 2020; Shanghan Lun) and passed through sieve No. 4. The samples were then dispersed into an aqueous solution for 2 h and decocted for 3 rounds of 4 h each. The aqueous solution was collected, concentrated and freeze-dried to obtain XCHT extract.

The XCHT extract was prepared by dissolving the sample in 80% methanol and subjecting it to sonication for 20 min using a KQ-500DA ultrasonic cleaner (Kunshan Ultrasonic Instruments Co., Ltd., Kunshan, China) at 40 kHz, 500 W, at room temperature. The resulting solution was filtered through a 0.22 μm microporous filter membrane prior to analysis. The components of the XCHT extract were analyzed using a Shimadzu LC-30A HPLC system (Shimadzu Corporation, Kyoto, Japan), equipped with a Waters ACQUITY UPLC BEH C18 column (130 Å, 1.7 µm, 2.1 × 100 mm; Waters Corp., Milford, MA, USA; Cat. No. 186002352), maintained at 40 °C. The mobile phases consisted of 0.1% aqueous formic acid (A) and acetonitrile (B), with a flow rate set at 0.25 mL/min and an injection volume of 2 μL. The gradient elution program was as follows: 0–0.01 min, 5% B; 0.01–10 min, 5–30% B; 10–15 min, 30–95% B; 15–17 min, 95% B; 17–23.1 min, 95–5% B.

### 2.3. Anti-MASH Activity in Cells In Vitro

HepG2 (The cell line was obtained from the Cell Resource Center, Peking Union Medical College (PCRC), 1101HUM-PUMC000035) cells were maintained in low-glucose DMEM (Gibco, Thermo Fisher Scientific, Waltham, MA, USA) supplemented with 10% fetal bovine serum (FBS, Gibco, Thermo Fisher Scientific, Waltham, MA, USA) and 1% penicillin/streptomycin (Gibco, Thermo Fisher Scientific, Waltham, MA, USA) at 37 °C in a humidified atmosphere containing 5% CO_2_. The cells were cultured under optimal conditions to maintain a stable and healthy state and seeded at a density of 1 × 10^5^ cells/mL for subsequent biochemical assays. In vitro anti-MASH activity assays were performed using XCHT at 30, 60, and 120 μg/mL. At the same time, the widely used hepatoprotective drug silymarin (MedChemExpress, Monmouth Junction, NJ, USA, 30 μg/mL) in the MASH model was used as a positive control [12,13].

To induce steatosis, HepG2 cells were treated with free fatty acids (FFAs) at a final concentration of 1.5 mM to establish an in vitro model. The FFAs were prepared using a mixture of sodium oleate (Energy Chemical, Shanghai, China; OA) and sodium palmitate (Beijing Solarbio Science & Technology Co., Ltd., Beijing, China; PA) in a 2:1 ratio. Lipid accumulation was assessed by Oil Red O staining, and images were captured under a light microscope for qualitative analysis. For biochemical analysis, after cell collection and lysis, intracellular levels of triglycerides (TG, A110-1-1), total cholesterol (TC, A111-1-1), alanine aminotransferase (ALT, C009-2-1), aspartate aminotransferase (AST, C010-2-1), and glutathione (GSH, A006-2-1) were quantified using commercial assay kits (Nanjing Jiancheng, Nanjing, China). The intracellular production of reactive oxygen species (ROS) was assessed using an ROS assay kit (Beijing Solarbio Science & Technology Co., Ltd., Beijing, China; D6471), and ROS levels were quantified by flow cytometry using CytExpert software version 2.6 (Beckman Coulter Life Sciences, Indianapolis, IN, USA).

### 2.4. Anti-MASH Activities of XCHT in Mice

Male C57BL/6 mice (6 weeks old) were purchased from Hengrong Biotechnology (Tianjin, China). All animal experiments and procedures were conducted in accordance with national regulations. Mice were housed under specific pathogen-free (SPF) conditions with a 12 h light/dark cycle at 22 ± 2 °C and 55 ± 5% humidity, with free access to food and water. After one week of acclimation, the experiment was initiated.

After a one-week acclimatization period, the mice were randomly assigned into 6 groups (*n* = 6 per group): the Control group, which was fed a methionine-choline-sufficient (MCS) diet and treated with 200 μL of sodium carboxymethyl cellulose (CMC-Na) solvent; the Model group, which was fed a methionine-choline-deficient (MCD) diet and treated with 200 μL of CMC-Na solvent; the Positive drug group, which was fed an MCD diet and treated with silymarin (MedChemExpress, Monmouth Junction, NJ, USA, 50 mg/kg); and the XCHT extract groups, which were fed an MCD diet and treated with XCHT extract at doses of 15 mg/kg, 30 mg/kg, or 60 mg/kg.

Throughout the experiment, body weight was recorded every three days for a period of 5 weeks. At the conclusion of the study, blood samples, liver tissues, and intestinal contents were collected for biochemical analysis and histopathological examination of liver tissues. The experimental animal feed was obtained from Nantong Trophie Biotechnology Co., Ltd. (Nantong, China), and the assay kits were sourced from Nanjing Jiancheng Biotechnology Co., Ltd. (Nanjing, China). All procedures were performed in compliance with the guidelines outlined by the National Institutes of Health (NIH) for animal experimentation, and the animal protocols were approved by the Ethics Committee of Tianjin University of Science and Technology, China (approval No. 20231130-3).

### 2.5. Real-Time qPCR Analysis

Total RNA was extracted from mouse liver tissue using the Trizol reagent (Beijing Solarbio Science & Technology Co., Ltd., Beijing, China; Cat. No. R1100) according to the manufacturer’s protocol. The tissue samples were immediately snap-frozen and homogenized in liquid nitrogen prior to RNA extraction. Genomic DNA contamination was eliminated using the gDNA Eraser (Takara Bio Inc., Kusatsu, Shiga, Japan) before reverse transcription. For cDNA synthesis, the PrimeScript™ FAST RT Reagent Kit (Takara Bio Inc., Kusatsu, Shiga, Japan) was employed. The resulting cDNA was subsequently used for quantitative real-time PCR (qPCR) analysis. Quantitative PCR was performed using the TBGreen^®^ Premix ExTaq™II FAST (Takara Bio Inc., Kusatsu, Shiga, Japan). Primer information is shown in Table S1. All data were normalized to the expression of Gapdh as an internal control. Relative expression levels were calculated using the 2^−ΔΔCT^ method [14].

### 2.6. Western Blot Analysis

To assess the expression levels of relevant proteins in liver tissue, Western blotting was performed. Liver tissues were homogenized in RIPA lysis buffer (Beijing Solarbio Science & Technology Co., Ltd., Beijing, China; Cat. No. R0010) and the homogenates were centrifuged at 12,500 rpm for 20 min at 4 °C to collect the supernatant. The protein concentration was determined using the BCA assay (Beijing Solarbio Science & Technology Co., Ltd., Beijing, China; Cat. No. PC0020). Samples were then mixed with loading buffer and denatured. Protein separation was achieved via SDS-PAGE (sodium dodecyl sulfate-polyacrylamide gel electrophoresis), followed by transfer onto a PVDF membrane (Millipore, Billerica, MA, USA). The membrane was blocked with 5% non-fat milk in PBST (PBS containing 0.1% Tween-20, pH 7.4) and incubated with primary antibodies overnight at 4 °C. Afterward, the membrane was incubated with DyLight 680-conjugated goat anti-rabbit IgG (Abbkine, Wuhan, Hubei, China; Cat. No. A23720, 1:10,000) at room temperature for 1 h in the dark. Protein bands were visualized using an infrared laser imaging system (LI-COR Biosciences, Lincoln, NE, USA; Odyssey Clx), and quantification was performed with ImageJ (version 1.53t, National Institutes of Health, Bethesda, MD, USA). The antibodies used for Western blot were ACC1 (Affinity Biosciences, Changzhou, Jiangsu, China, AF6421, 1:1000), p-ACC (Affinity Biosciences, Changzhou, Jiangsu, China, AF7364, 1:1000), AMPK alpha (Affinity Biosciences, Changzhou, Jiangsu, China, AF6423, 1:1000), p-AMPK alpha (Affinity Biosciences, Changzhou, Jiangsu, China, AF3423, 1:1000), α-tubulin (Affinity Biosciences, Changzhou, Jiangsu, China, AF7010, 1:5000).

### 2.7. Gut Microbiota Analysis

At the conclusion of the experimental period, fecal samples were collected from all mice and immediately stored in liquid nitrogen to preserve the stability of the microbiota DNA. The gut microbiota was analyzed through 16S rRNA gene sequencing. DNA extraction from fecal samples was carried out according to the protocols outlined by BGI Genomics (Shenzhen, China).

### 2.8. Molecular Docking Verification Between the Core Substances and Key Targets

Protein structure files in PDB format were retrieved and downloaded from the RCSB PDB database. The structures were then dehydrogenated, hydrogenated, and prepared for protein receptor simulations using Maestro 13.5 (Schrödinger, Inc., New York, NY, USA). The structural formulas of the key components of XCHT, identified through network pharmacology, were drawn using ChemBioDraw Ultra 14.0 (PerkinElmer, Inc., Waltham, MA, USA. These small molecules were subsequently converted into PDB format using ChemBio3D 14.0 (PerkinElmer, Inc., Waltham, MA, USA). The preparation of the small molecules, the generation of docking boxes, and the docking of small molecules with protein receptors were performed using Maestro 13.5. Docking results were imported into Discovery Studio 4.5 Client (BIOVIA, Dassault Systèmes, San Diego, CA, USA) for 2D visualization of the docking interactions, and were also analyzed in PyMOL Version 2.6 (Schrödinger, LLC, New York, NY, USA) for molecular docking visualization.

### 2.9. Data Analysis Statistics

GraphPad Prism version 9.0 (GraphPad Software, San Diego, CA, USA) and SPSS version 27.0 (IBM Corp., Armonk, NY, USA) were used for statistical analysis. The experimental data were shown as the mean ± SEM. The results were analyzed by one-way analysis of variance (ANOVA). *p* < 0.05 was considered significant.

## 3. Results

### 3.1. Primary Active Ingredients and Targets of XCHT Associated with MASH

**Figure 1:**
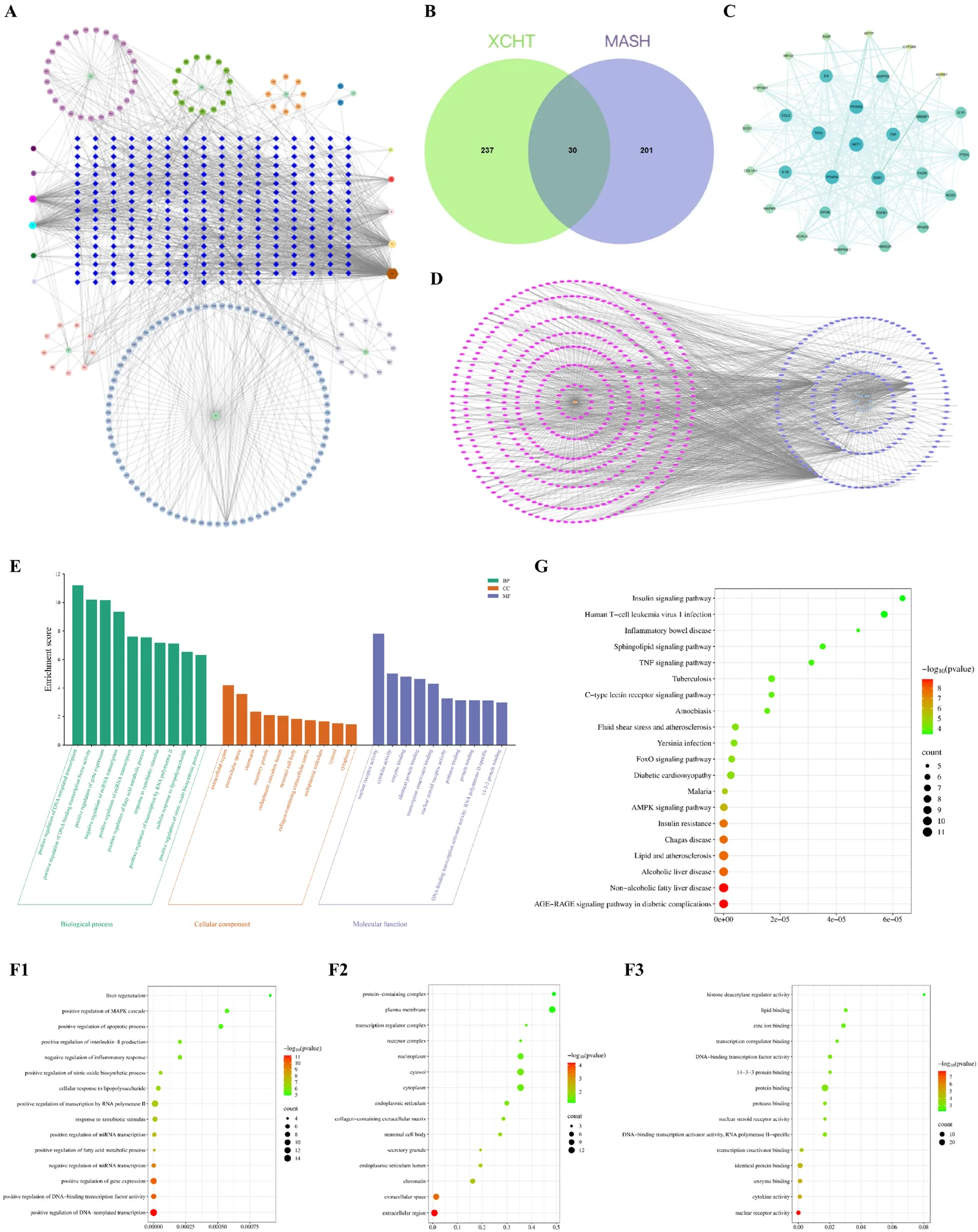
Network pharmacology-based analysis. (**A**) Herb–ingredient–target gene network. (**B**) The Venn diagram of XCHT-MSAH. (**C**) The protein–protein interaction (PPI) network of XCHT in the treatment of MASH. (**D**) Drug–active ingredient–common target–disease network. (**E**,**F**) GO enrichment analysis of common targets. ((**F1**): The GO-BP enrichment analysis. (**F2**): The GO-CC enrichment analysis. (**F3**): The GO-MF enrichment analysis). (**G**) KEGG enrichment analysis of common targets.

The TCMSP database was used to screen the active compounds (OB ≥ 30%, DL ≥ 0.18) of XCHT, and 214 active compounds were obtained, and 167 active compounds were retained after excluding those with no corresponding targets, which corresponded to 267 potential targets, constructing the “herbal medicine–component–target” network (Figure 1A).

Integration of GeneCards, OMIM and DrugBank databases obtained 231 MASH-related targets. Intersections were taken with XCHT targets and 30 common targets were identified (Figure 1B) as potential targets of action for XCHT for MASH. The PPI network was constructed using the STRING database, and the core target was located at the center of the network (Figure 1C). The “drug–component–target–disease” network was further constructed, and the top 10 active ingredients were screened for their topological properties (Table S2).

GO analysis showed that the targets were significantly enriched in the processes of gene expression regulation, fatty acid metabolism and apoptosis (Figure 1E,F). KEGG pathway analysis identified 106 significant pathways, the top 20 of which were involved in inflammation and hormone metabolism (Figure 1G), among which the TNF, MAPK and AMPK signaling pathways were closely related to the pathogenesis of MASH, suggesting that they were the key action pathways of XCHT.

### 3.2. Qualitative Analysis of XCHT

**Figure 2:**
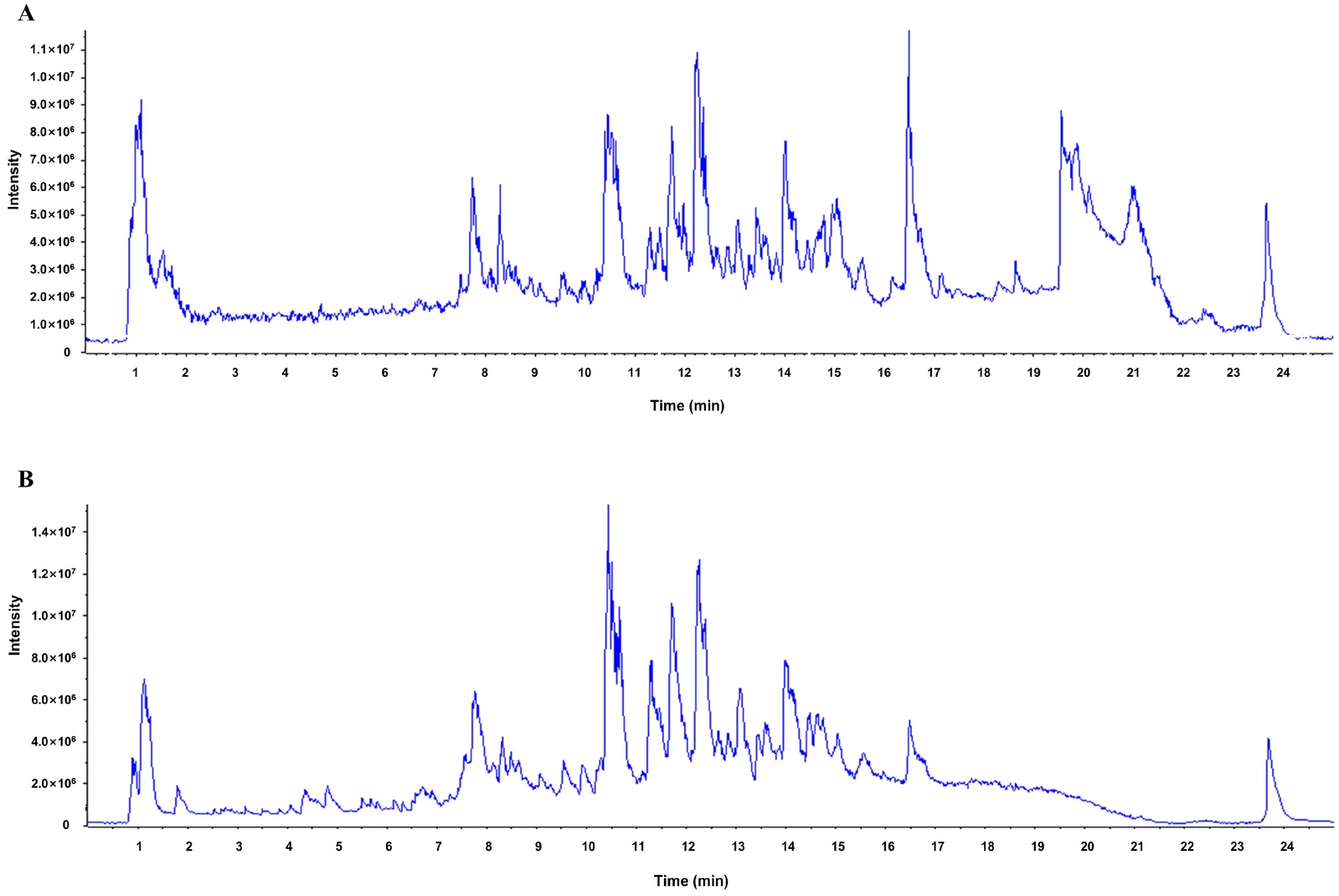
UHPLC-QTOF-MS was used to qualitatively analyze XCHT. The structure identification was carried out and 20 compounds (10 positive ion (**A**) and negative ion compounds (**B**)) were identified by UHPLC-QTOF-MS.

XCHT was qualitatively analyzed using UHPLC-QTOF-MS to obtain a total chromatogram. Most of the chemical constituents of XCHT could be detected within 1–25 min (Figure 2).

The MS/MS fragmentation pattern was the main evidence for compound identification. The structures were identified to obtain 171 compounds by comparison with database information and available literature based on similarity in mass spectral behavior and retention time (Table S3). The types of compounds identified mainly included flavonoids, flavonoid glycosides, and saponins.

### 3.3. XCHT Improves Hepatocyte Injury and Lipid Accumulation

**Figure 3:**
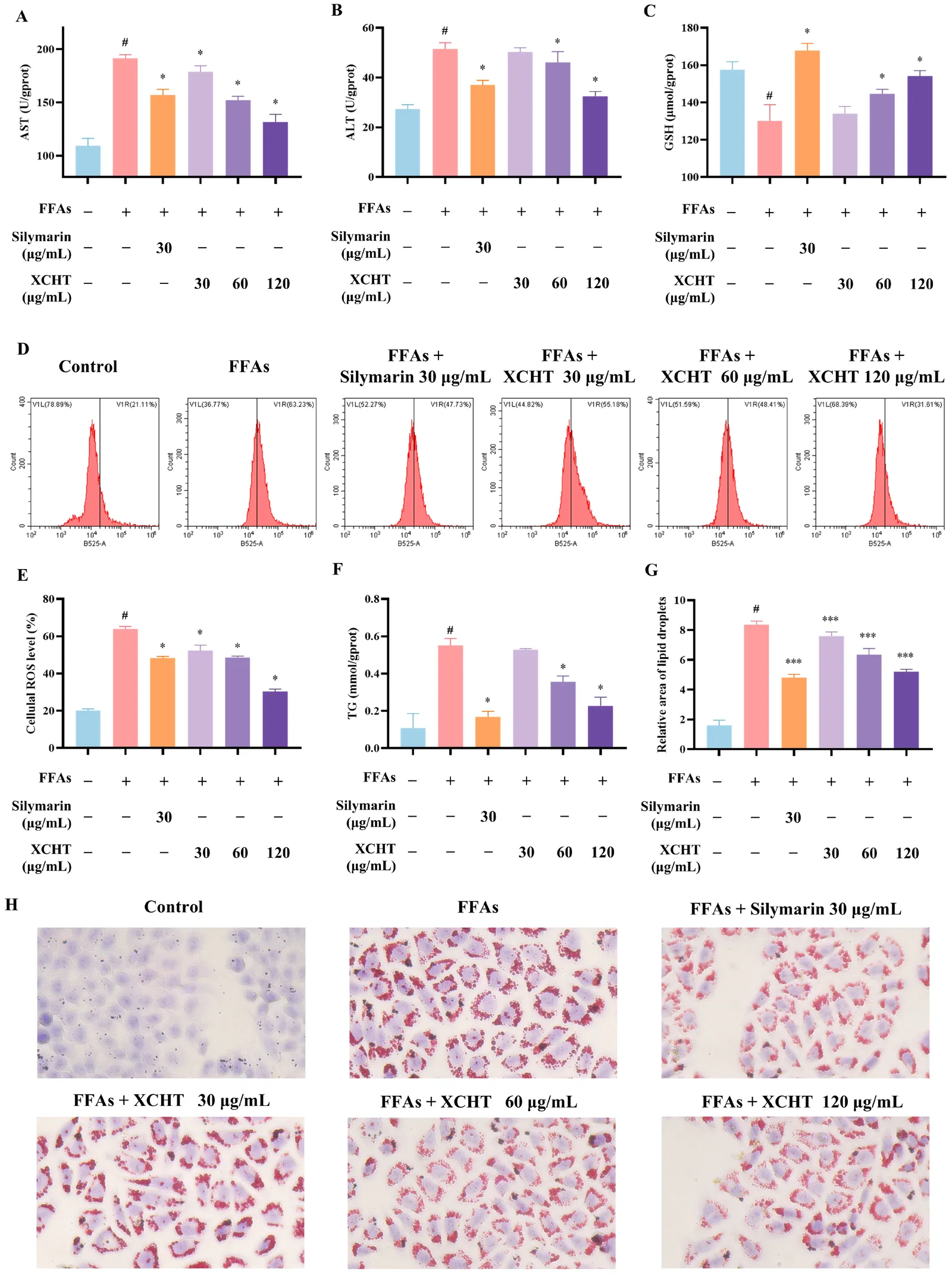
Effects of XCHT on lipid accumulation in HepG2 cells. XCHT alleviated FFA-induced HepG2 cell damage, reduced AST (**A**) and ALT (**B**) activities, elevated GSH (**C**) content, decreased ROS generation (**D**) and ROS levels (The vertical line indicates the gating threshold used to define the ROS-positive cell population.) (**E**), and alleviated lipid accumulation, as shown by reduced TG (**F**), decreased lipid accumulation (**G**), and Oil Red O staining (**H**). # *p* < 0.05 vs. Control group. * *p* < 0.05, *** *p* < 0.001 vs. Model. n = 3 per group.

To investigate the effects of XCHT on hepatocyte injury and lipid accumulation under metabolic stress, we stimulated HepG2 cells with free fatty acids (FFAs, 1.5 mM) and treated the cells with XCHT extract (30, 60, and 120 μg/mL) for 24 h. The results showed that FFAs induced elevated AST (Figure 3A) and ALT (Figure 3B) activities in HepG2 cells, suggesting that excessive lipid accumulation may contribute to hepatocyte injury. In contrast, XCHT-treated HepG2 cells stimulated with FFAs showed a significant reduction in AST and ALT activities, indicating that XCHT attenuates hepatocyte injury and reduces susceptibility to lipid accumulation. In addition, FFA treatment induced oxidative stress and lipid peroxidation, as evidenced by decreased GSH (Figure 3C) levels and increased ROS production (Figure 3D,E). These pathological responses were significantly alleviated by XCHT treatment, indicating that XCHT enhanced the antioxidant capacity of hepatocytes and attenuated oxidative stress-induced cellular damage and dysfunction. The inhibitory effect of XCHT on intracellular lipid accumulation was further verified by Oil Red O staining (Figure 3H), and the quantitative analysis was consistent with this observation (Figure 3G). Furthermore, measurement of triglyceride (TG) content (Figure 3F) showed that XCHT treatment significantly reduced lipid accumulation, further confirming the beneficial effect of XCHT in attenuating lipid accumulation in hepatocytes.

### 3.4. XCHT Improves In Vivo Liver Injury and Inflammatory Response in MCD-Induced MASH Mice

**Figure 4:**
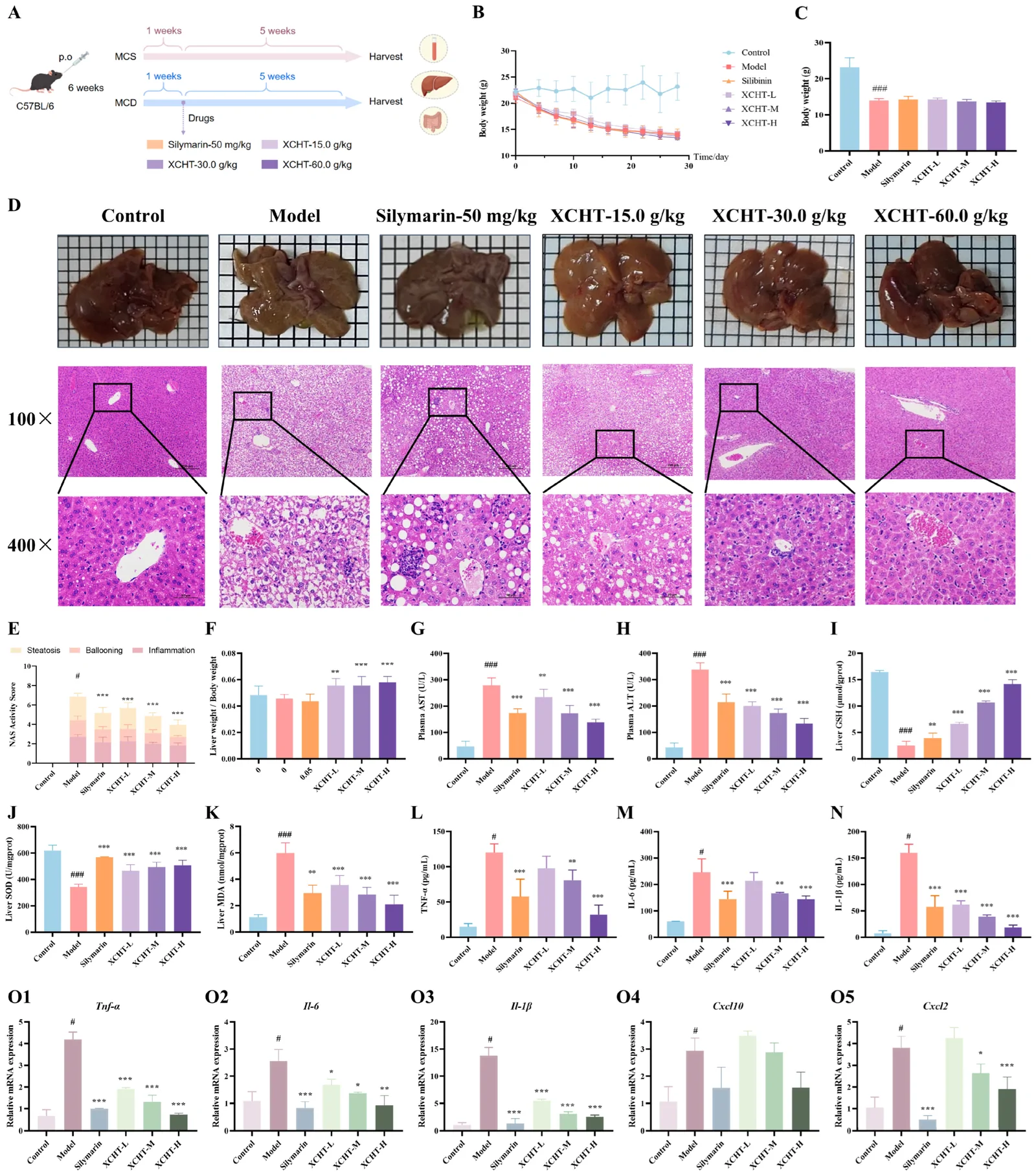
Effect of XCHT on liver injury and inflammatory response in MASH mice. The experiments were performed according to established animal experimental procedures (**A**). Changes in body weight of mice during the 5-week MCD diet (**B**), and body weight of mice on the last day (**C**). XCHT had a protective effect against liver injury in MASH mice. XCHT improved the pathological changes in the liver (**D**) and NAFLD activity score (NAS) (**E**), which was calculated as the sum of steatosis (0–3), lobular inflammation (0–3), and hepatocyte ballooning (0–2) (total score 0–8). XCHT also improved liver weight (**F**). Meanwhile, XCHT significantly reduced the plasma activities of alanine transaminase (AST) (**G**) and alanine transaminase (ALT) (**H**). Liver levels of GSH (**I**), SOD (**J**), and MDA (**K**) were altered by MCD, and XCHT altered their expression. In addition, plasma levels of TNF-α (**L**), IL-6 (**M**), and IL-1β (**N**) were elevated in MASH mice, whereas their expression was significantly reduced with XCHT. At the mRNA level, XCHT significantly regulated the expression of inflammation-related genes (**O1**–**O5**). # *p* < 0.05, ### *p* < 0.001 vs. Control group. * *p* < 0.05, ** *p* < 0.01, *** *p* < 0.001 vs. Model. n = 6 per group.

To further explore the protective effect of XCHT on liver injury, we investigated whether XCHT could alleviate liver injury in mice fed an MCD diet (Figure 4A). MCD diet mice showed significant weight loss compared to normal diet control mice and continued to lose weight over time (Figure 4B,C). Histological analysis by hematoxylin-eosin (HE) staining (Figure 4D) and NAS activity score (Figure 4E) showed that the livers of MCD mice showed significant damage, including a series of pathological features such as ballooning, inflammatory cell infiltration, and diffuse lipoatrophy. In contrast, XCHT treatment significantly attenuated MCD-induced liver injury in MCD mice. After 5 weeks of MCD feeding, the liver weights of MCD diet mice were significantly decreased, whereas the liver weights of mice in the XCHT-treated group recovered in comparison to the MCD group (Figure 4F). In addition, plasma AST (Figure 4G) and ALT (Figure 4H) activity measurements showed that the plasma AST and ALT activities of XCHT-treated MCD-fed mice were significantly lower than those of the MCD dietary model group, further suggesting that XCHT was able to alleviate MCD-induced liver injury.

In the liver tissues of MCD diet mice, we observed a significant decrease in SOD and GSH content, along with a significant increase in lipid peroxides MDA content, suggesting that enhanced oxidative stress in the liver leads to increased liver cell injury (Figure 4I–K). However, after XCHT treatment, the level of oxidative stress was significantly alleviated in the liver of mice, and SOD and GSH levels were significantly elevated, whereas MDA content was significantly reduced. The pro-inflammatory cytokines TNF-α (Figure 4L), IL-6 (Figure 4M), and IL-1β (Figure 4N) were significantly elevated in MCD dietary mice, whereas the levels of pro-inflammatory factors were significantly reduced after XCHT extract treatment. In addition, real-time quantitative PCR results showed (Figure 4O1–O5) that XCHT significantly inhibited the expression of genes related to inflammatory response, such as *Tnf-α*, *Il-6*, *Il-1β*, *Cxcl10*, and *Cxcl2*, in the liver of MCD diet mice.

In summary, XCHT significantly ameliorated liver injury and inflammatory responses in MCD-fed mice, possibly exerting its protective effects by inhibiting oxidative stress and inflammatory responses.

### 3.5. XCHT Improves In Vivo Lipid Accumulation in MCD-Induced MASH Mice

**Figure 5:**
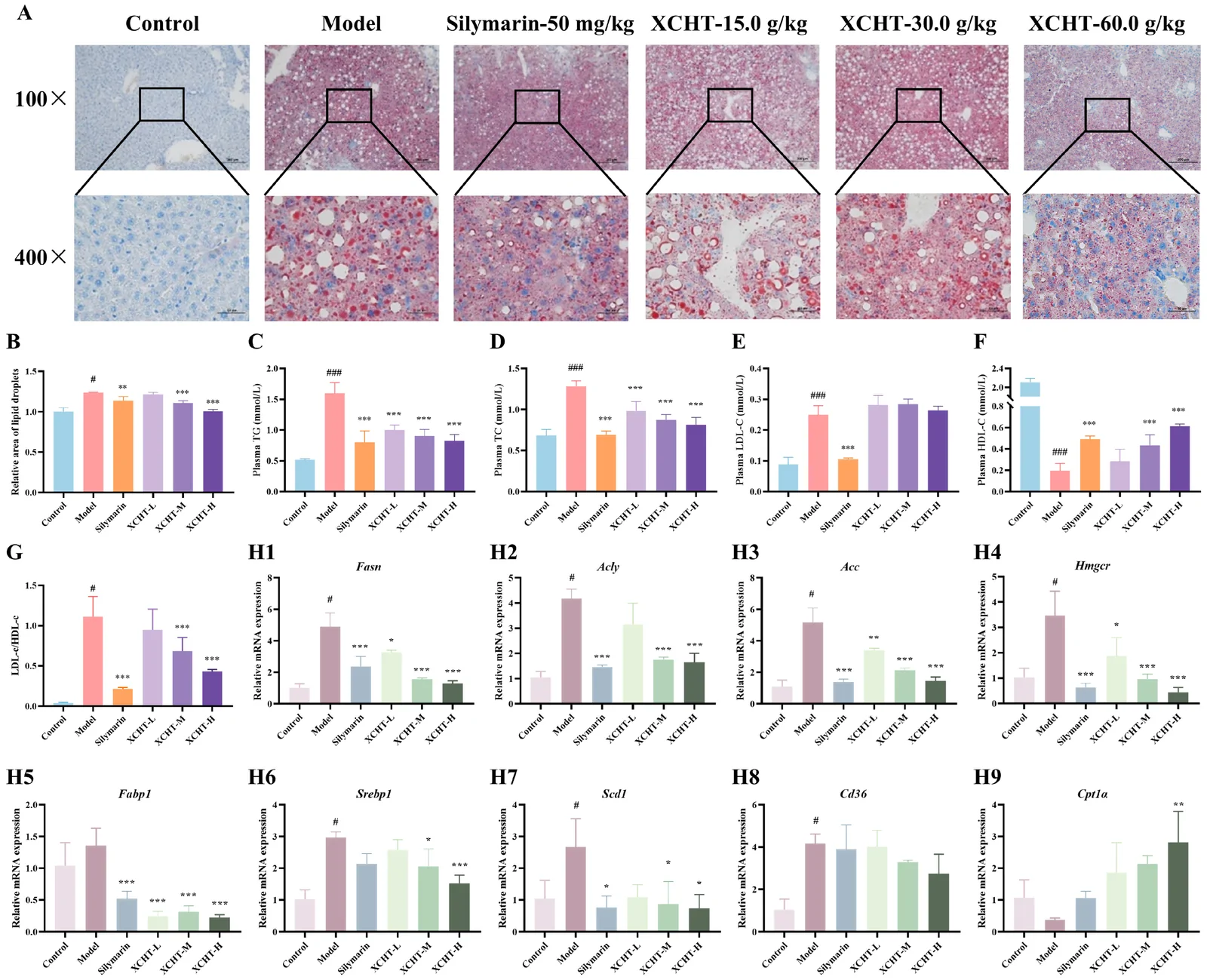
Effect of XCHT on lipid accumulation in MCD mice. Oil red O staining (**A**) and quantitative analysis (**B**) suggested that XCHT significantly reduced the accumulation of lipid droplets in the liver tissue of mice. In the MCD-induced MASH model, the levels of TG (**C**), TC (**D**), and LDL-c (**E**) were significantly elevated, the content of HDL-c was significantly reduced (**F**), and the ratio of LDL-c/HDL-c (**G**) was significantly altered, whereas a significant modulatory effect of XCHT on their expression was observed. In addition, XCHT significantly affected the expression of lipid metabolism-related genes in liver tissue (**H1**–**H9**). # *p* < 0.05, ### *p* < 0.001 vs. Control group. * *p* < 0.05, ** *p* < 0.01, *** *p* < 0.001 vs. Model. n = 6 per group.

The results of liver Oil Red O staining (Figure 5A) and quantitative analysis (Figure 5B) showed that XCHT treatment significantly inhibited the accumulation of lipid droplets in the livers of MCD diet mice. Plasma TG and TC levels were significantly decreased in the XCHT-treated group of mice compared with the control MCD mice (Figure 5C,D). The lipoproteins LDL-c (Figure 5E) and HDL-c (Figure 5F), which are associated with cholesterol transport, were further analyzed. LDL-c levels were significantly elevated and HDL-c levels were significantly decreased in MCD diet mice. XCHT treatment successfully reversed this change, and the LDL-c/HDL-c ratio (Figure 5G) was significantly lower than that in the MCD diet group.

TG-rich lipid droplets in liver cells are a typical hallmark of MASH, and multiple genes associated with lipogenesis, fatty acid uptake and oxidation regulate this process. To further explore the effect of XCHT on lipid metabolism, we examined the expression of key genes related to hepatic fatty acid synthesis and uptake by real-time quantitative PCR. The results showed that the expression of lipogenesis and uptake-related genes such as *Fasn*, *Acly*, *Acc*, *Hmgcr*, *Fabp1*, *Srebp1*, *Scd1*, and *Cd36* was significantly increased in the MCD diet group, while the expression of *Cpt1α* was significantly decreased, and the XCHT treatment group succeeded in reversing the changes in the expression of these genes (Figure 5H1–H9).

### 3.6. XCHT Improves MCD-Induced Gut Microflora Disruption in MASH Mice

**Figure 6:**
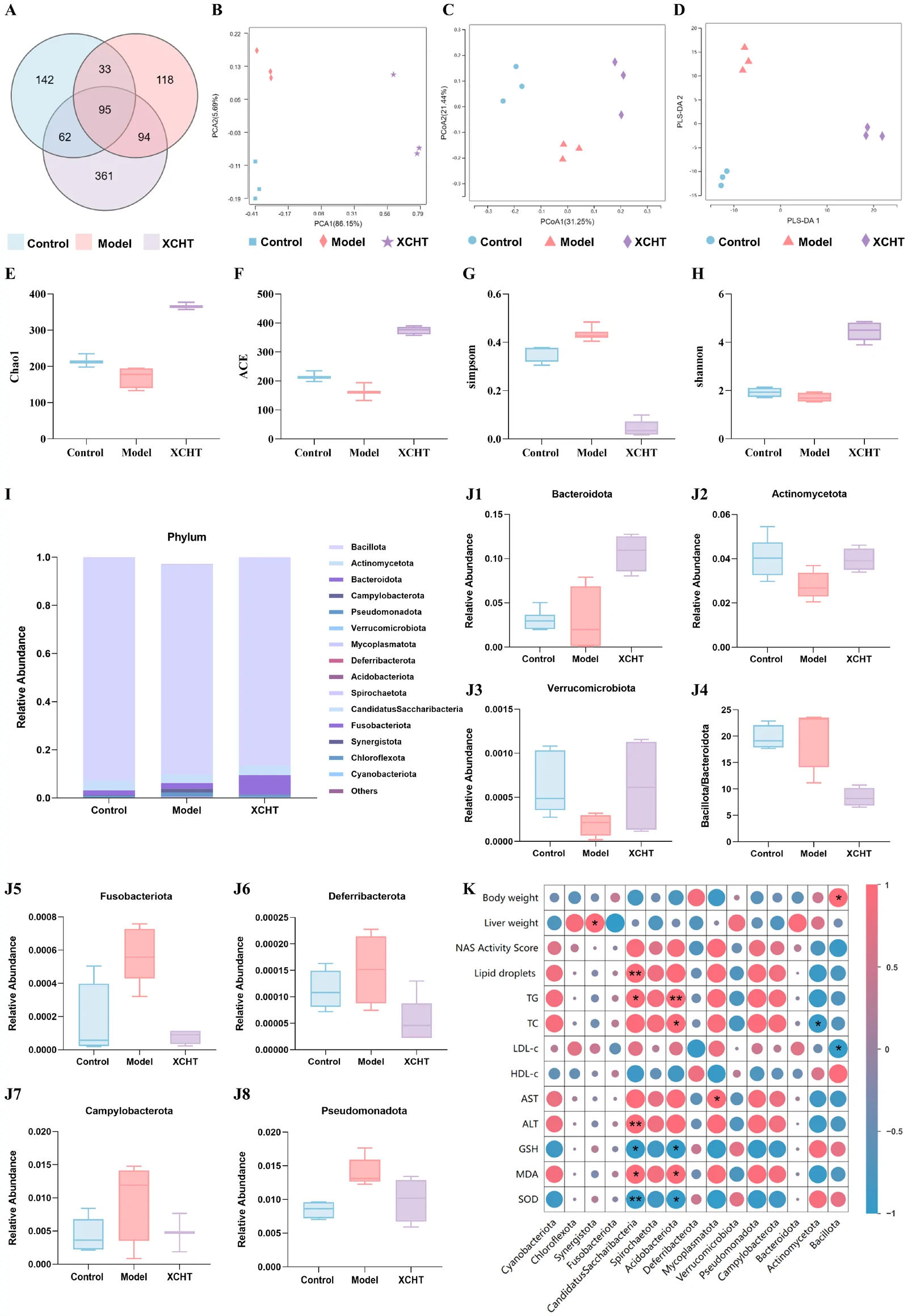
Effects of XCHT on the intestinal flora. Venn diagram of XCHT (**A**). PCA (**B**), PCoA (**C**), and PLS-DA (**D**) analyses of XCHT. Alpha diversity of XCHT (**E**–**H**). Species abundance at the XCHT portal level (**I**), and corresponding bacterial flora analysis (**J1**–**J8**). Correlation analyses of species at the XCHT portal level with the Pearson correlation analysis of MASH disease (**K**). * *p* < 0.05, ** *p* < 0.01.

The gut microbiota plays a crucial role in the pathogenesis of MASH, and its intervention may be a viable therapeutic strategy. We analyzed 16S rDNA gene sequences of colonic contents to assess structural changes in the gut microbiota of MASH mice. The Venn diagram showed (Figure 6A) that a total of 905 operational taxonomic units (OTUs) were identified in all samples, with a total of 95 OTUs between different experimental groups. 142, 118, and 361 different OTUs were found in the Control, Model, and XCHT groups, respectively. PCA analysis (Figure 6B) reflected the β-diversity and revealed the changes in the composition of the gut microbiota between the MCD and XCHT groups. The PCA results showed that there was a tendency of some dispersion between the groups and a more aggregated tendency within the groups. Further PCoA analysis (Figure 6C) demonstrated different clustering patterns among the gut microbiota of each experimental group. Both MCD and XCHT groups resulted in significant changes in microbial community structure. PLS-DA analyses (Figure 6D) showed a significant separation of gut microbiota among the three groups of samples. The alpha diversity indices Chao1 (Figure 6E) and ACE (Figure 6F) were significantly increased in the XCHT group compared to the MCD group, indicating that the abundance of the gut microbiota was enhanced. In addition, Simpson’s index (Figure 6G) decreased and Shannon’s index (Figure 6H) increased, indicating an increase in the diversity of the gut microbiota after XCHT treatment. These results suggested that XCHT treatment may help to restore the loss of gut microbial diversity due to MCD.

To assess the effect of XCHT on the structure of the gut flora, the abundance of the gut microbiota at the phylum level was further analyzed (Figure 6I), and Bacillota and Bacteroidota were the main dominant phyla in each group, with more than 80% of the abundance. Relative to the MCD group, XCHT treatment significantly increased the abundance of Bacteroidota, Actinomycetota, and Verrucomicrobiota (Figure 6J1–J3). At the same time, the Bacillota/Bacteroidota ratio decreases (Figure 6J4), while the abundance of Fusobacteriota, Deferribacterota, Campylobacterota, and Pseudomonadota was significantly decreased (Figure 6J5–J8). A Pearson correlation analysis of the correlation between MASH biomarkers and gut microbiota showed a significant negative correlation between Actinomycetota and TC (Figure 6K).

In conclusion, XCHT treatment was effective in reversing MCD diet-induced gut microbiota dysbiosis and improved MASH symptoms.

### 3.7. Key Targets and Molecular Docking of XCHT for the Treatment of MASH

**Figure 7:**
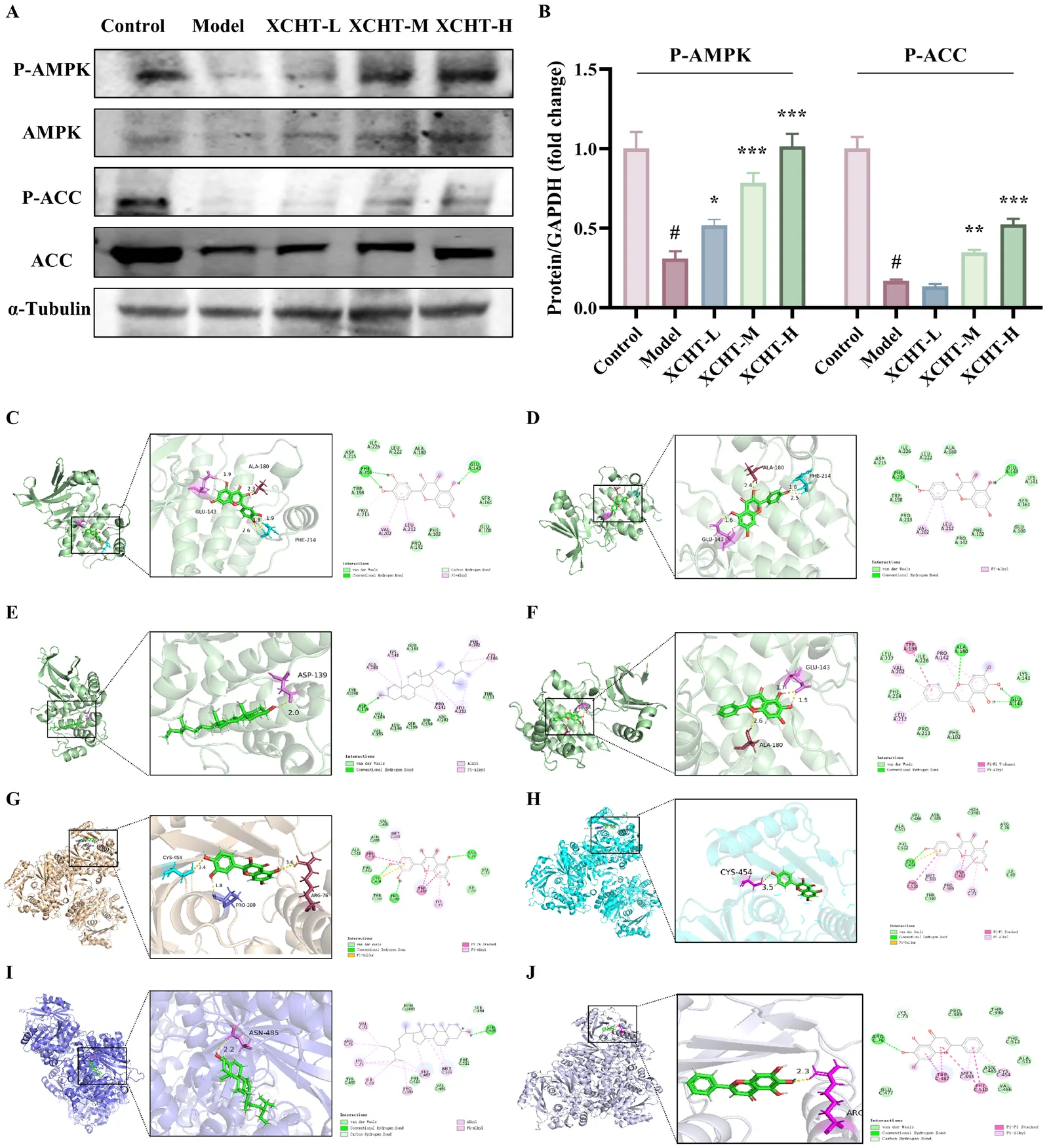
Key targets and molecular docking of XCHT for the treatment of MASH. XCHT significantly increased AMPK, ACC protein phosphorylation (**A**,**B**). Molecular docking of Quercetin (**C**), Kaempferol (**D**), β-Sitosterol (**E**), Baicalein (**F**), key active components of XCHT, with AMPK. And molecular docking of Quercetin (**G**), Kaempferol (**H**), β-Sitosterol (**I**), Baicalein (**J**) with ACC. # *p* < 0.05, vs. Control group. * *p* < 0.05, ** *p* < 0.01, *** *p* < 0.001 vs. Model.

Network pharmacological analysis has shown that the AMPK pathway is a key pathway for XCHT in the treatment of MASH. The core kinases in the AMPK pathway are closely associated with targets in a variety of pathological processes such as lipid metabolism, inflammatory responses, and fibrosis [15]. AMPK is a sensor of cellular energy status and is present in heterotrimeric complexes, including the catalytic α-subunit and the regulatory β-subunit, as well as the γ-subunit [16]. AMPK regulates cellular energy metabolism by phosphorylating other downstream target proteins. ACC is a major substrate of AMPK, and the phosphorylation status of ACC is often used as a marker of AMPK activation. Therefore, to investigate whether XCHT exerts a therapeutic effect on MASH by regulating AMPK activity, we examined the levels of p-AMPK and p-ACC (Figure 7A,B). The results showed that XCHT treatment significantly increased the levels of p-AMPK and p-ACC in mouse liver tissue. It is suggested that XCHT plays a role in MASH treatment by regulating the AMPK/ACC signaling pathway.

Network pharmacological analysis revealed that four compounds, quercetin, kaempferol, β-sitosterol and baicalein, played important roles in the treatment of MASH. To delve into the binding mechanisms of these key active ingredients to their targets, we performed molecular docking analysis (Figure 7C–J) to assess the binding affinities of these compounds to AMPK and its related targets. The molecular docking score (kcal/mol) was used to assess the strength of compound binding to the target. In the present study, all the binding energy values were below −2.888 kcal/mol, ranging from −7.146 to −2.888, indicating that the four compounds exhibited moderate to strong binding activity to AMPK and ACC.

## 4. Discussion

**Figure 8:**
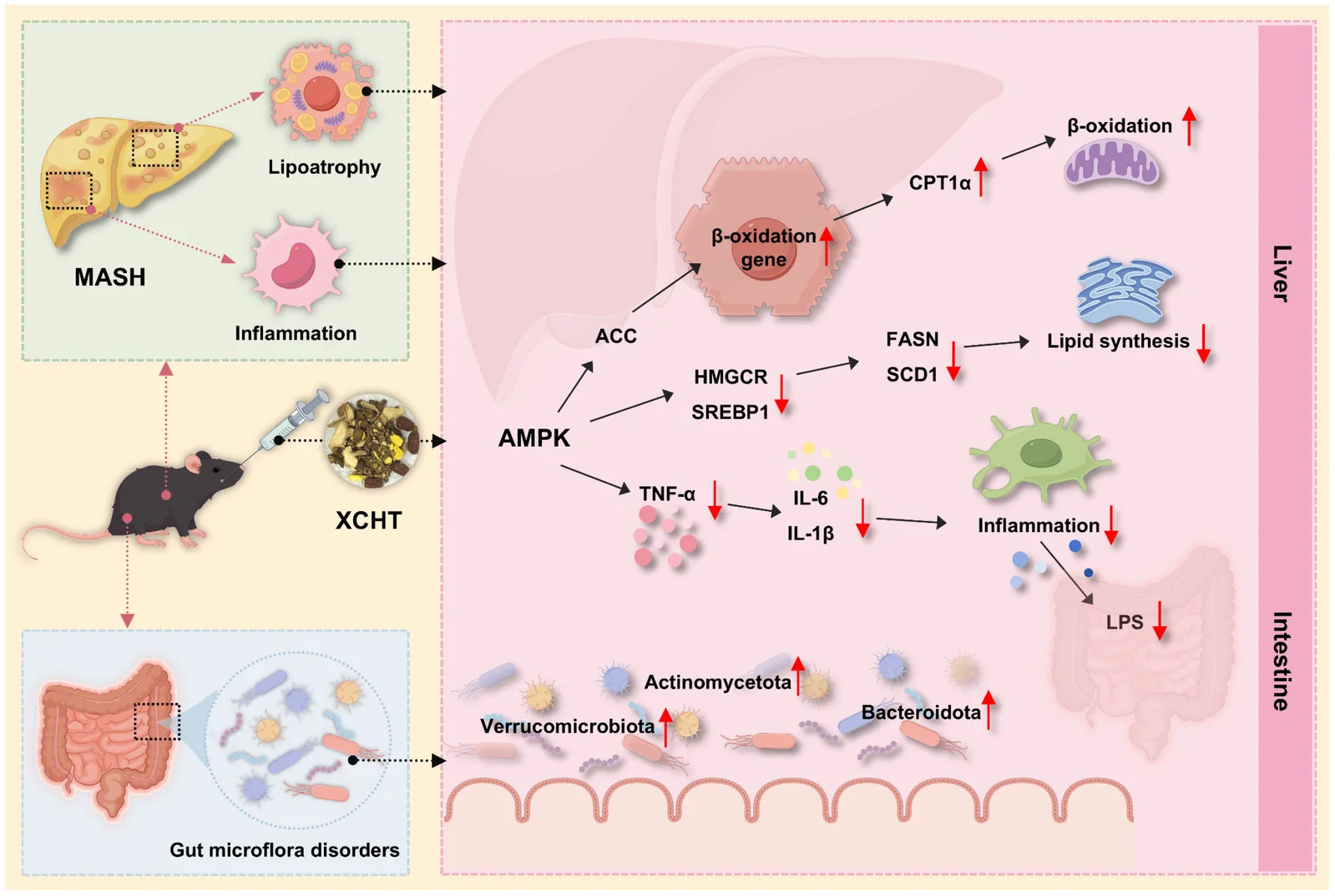
A proposed schematic model of the possible underlying molecular mechanisms associated with the therapeutic effect of XCHT against MASH. Black arrows indicate the sequence of events; red arrows indicate increases (upward) and decreases (downward).

In this study, we systematically investigated the multi-target therapeutic potential of XCHT in MASH, revealed its role in ameliorating liver cell injury, lipid metabolism disorders, oxidative stress, and intestinal flora dysbiosis, and elucidated the underlying mechanisms through network pharmacology and molecular docking techniques (Figure 8).

As a traditional formula, XCHT has accumulated clear experimental data and significant clinical effects in the treatment of liver-related diseases [17,18]. In this study, we analyzed and identified the components of XCHT and found that it regulates the progression of MASH through the synergistic effect of multiple components. Qualitative analysis using UHPLC-QTOF-MS identified the characteristic components of traditional Chinese medicine in XCHT, including flavonoids, saponins and organic acids. A comprehensive network pharmacology analysis suggests that quercetin, kaempferol, β-sitosterol, and baicalin may be the primary active components in XCHT for the treatment of MASH; they exert synergistic antioxidant effects and play a role in inhibiting MASH. These compounds may modulate the pathological process of MASH by synergistically regulating core pathways such as AMPK/ACC. Specifically, quercetin has pro-autophagic and anti-inflammatory effects by preventing MASLD through AMPK-mediated mitochondrial autophagy and reducing intracellular lipid and ROS accumulation [19]. Kaempferol regulates hepatic lipid accumulation through activation of Sirt1/AMPK signaling, ameliorates hepatic injury, and regulates the expression of fatty acid oxidation-related proteins, while down-regulating the level of the lipid synthesis-related protein ACC [20]. β-sitosterol has beneficial effects on glucose and lipid metabolism, significantly increasing phosphorylation of AMPK and ACC, promoting glucose uptake and modulating lipid metabolism [21]. Baicalein ameliorates MASLD-associated biochemical abnormalities by decreasing lipid accumulation through the activation of AMPK and inhibiting SREBP1 cleavage [22]. It also exhibits significant attenuation of liver injury and anti-inflammatory effects [23]. Therefore, the discovery of these compounds provides a scientific basis for the active ingredients in MASH therapy and helps to further elucidate their mechanisms of action and targets.

AMPK, as a core regulator of energy metabolism, is involved in the regulation of various cellular processes such as lipid metabolism, glucose metabolism, and redox reactions [16,24]. In MASH, the bacterial signaling molecule lipopolysaccharide [25] and inflammatory cytokines such as TNF-α inhibit AMPK activity, thereby reducing mitochondrial biotransformation and mitochondrial autophagy. The small molecule PXL770 activates AMPK and increases mitochondrial function and fatty acid oxidation, while inhibiting fat de novo synthesis (DNL), which together reduce steatosis and fibrosis in mice [26]. Furthermore, clinical studies have shown that AMPK activation inhibits DNL [27] and attenuates steatosis in type 2 diabetic patients [28]. Of interest, thyroid hormone also activates AMPK in liver cells, an effect that is strongly associated with fatty acid oxidation and increased mitochondrial autophagy [29]. Therefore, in conjunction with these data, it is hypothesized that some of the beneficial effects of Resmetirom, the only FDA-approved MASH drug targeting thyroid receptor ß, may be mediated through the AMPK pathway. Based on this, drugs targeting AMPK show important potential in MASH therapy. Our findings support this view. AMPK phosphorylation levels were significantly reduced in liver tissues of MCD diet-induced MASH mice. In contrast, XCHT significantly up-regulated the expression of p-AMPK and p-ACC, suggesting that XCHT is able to act as an AMPK agonist to activate the AMPK signaling pathway, inhibit lipid synthesis and promote fatty acid oxidation, thereby ameliorating MASH. AMPK inhibits the production of malonyl-coenzyme A by phosphorylating ACC and deregulates the inhibition of CPT1α, thereby promoting mitochondrial β-oxidation [30]. This is consistent with the upregulation of Cpt1α expression and decrease in TG/TC levels after XCHT treatment. In addition, the activation of AMPK attenuated the inflammatory response by inhibiting the NF-κB pathway, which echoed the down-regulation of pro-inflammatory factors such as TNF-α and IL-6. In conclusion, the potential of XCHT as an AMPK agonist in the treatment of MASH warrants further investigation.

In the present study, it was revealed for the first time that XCHT could reverse the MCD-diet-induced intestinal dysbiosis through the liver–intestinal axis, which was manifested as an increase in the abundance and diversity of intestinal flora and an alteration in the structure of intestinal flora in mice. At the portal level, the ratio of Bacillota/Bacteroidota in the XCHT group was decreased and the abundance of the Bacteroidota phylum was increased compared to the MCD group. Bacillota is a harmful bacterium, and its main metabolites, acetic and propionic acids, are the substrates for hepatic gluconeogenesis and lipogenesis [31]. The Bacillota/Bacteroidota ratio is higher in MASLD patients and obese bodies, resulting in increased lipopolysaccharide (LPS) production and disruption of intestinal immune homeostasis [32]. The abundance of Actinomycetota is positively correlated with physical fitness [33], which can promote energy metabolism and modulate hepatic lipid synthesis and inflammatory responses to prevent MASH progression and maintain gut flora homeostasis [34]. The negative correlation between Actinomycetota and TC in the present study suggested that it may alleviate hepatic injury through anti-inflammatory effects. Metabolites of the gut microbiota, such as short-chain fatty acids and LPS, are key factors influencing oxidative stress in the liver. Among these, LPS can activate hepatic NADPH oxidase via the Toll-like receptor 4 (TLR4) pathway and induce a burst of ROS [35]. Therefore, XCHT-mediated gut microbiota remodeling can be regarded as an indirect yet sustained intestinal antioxidant process. This synergistic interaction between direct activation of hepatic AMPK and indirect regulation by the gut microbiota constitutes the network through which XCHT regulates the redox state of MASH [36].

This study demonstrates that XCHT ameliorates MCD-diet-induced MASH in mice, as evidenced by reduced hepatic injury and lipid accumulation, modulated gut microbiota composition, and activation of the AMPK/ACC pathway. Network pharmacology predicted quercetin, kaempferol, β-sitosterol, and baicalin as key active components of XCHT. These findings suggest that XCHT may exert its therapeutic effects on MASH through multi-target regulation of hepatic redox balance, lipid metabolism, and gut microbiota, potentially involving AMPK/ACC signaling and the gut–liver axis. However, the precise contributions of each active component and the causal role of LPS-mediated oxidative damage and the gut–liver axis in mediating the effects of XCHT require further experimental validation. Future studies should integrate metabolomics and single-cell sequencing to comprehensively analyze the “component–microbiota–metabolism–immunity” network through which XCHT regulates MASH.

## Data Availability

The original contributions presented in this study are included in the article/Supplementary Material. Further inquiries can be directed to the corresponding author.

## Supplementary Materials

The following supporting information can be downloaded at: https://www.mdpi.com/article/10.3390/antiox15101268/s1, Table S1: The primer sequence of target gene; Table S2: Topological properties of the top 10 active ingredients; Table S3: Information of the positive ion compounds from XCHT tentatively characterized by UHPLC-QTOF-MS.

## Abbreviations

ACC, acetyl CoA carboxylase; ACE, Abundance-based Coverage Estimator; *Acly*, ATP citrate lyase; ALT, alanine aminotransferase; AMPK, AMP-activated protein kinase; AST, aspartate aminotransferase; BP, biological processes; CC, cellular components; *Cd36*, cluster of differentiation 36; cDNA, complementary DNA; CMC-Na, Carboxymethyl Cellulose Sodium; *Cpt1α*, carnitine palmitoyltransferase 1α; *Cxcl10*, C-X-C motif chemokine ligand 10; *Cxcl2*, C-X-C motif chemokine 2; DL, drug-likeness; *Fabp1*, fatty acid binding protein 1; *Fasn*, fatty acid synthase; FBS, fetal bovine serum; FFAs, free fatty acid; *Gapdh*, glyceraldehyde-3-phosphate dehydrogenase; gDNA, genomic DNA; GO, gene ontology; GSH, glutathione; HDL-c, high-density lipoprotein cholesterol; HE, hematoxylin-eosin staining; HFD, high-fat diet; *Hmgcr*, 3-hydroxy-3-methylglutaryl-CoA reductase; HSCs, hepatic stellate cells; IgG, Immunoglobulin G; IL-1β, Interleukin-1 beta; IL-6, interleukin-6; KEGG, kyoto encyclopedia of genes and genomes; LDL-c, low-density lipoprotein cholesterol; LPS, lipopolysaccharide; MASH, metabolic dysfunction-associated steatohepatitis; MASLD, Metabolic dysfunction-associated steatohepatitis; MCD, methionine-and choline-deficent diet; MCS, methionine-and choline-supplement diet; MDA, malondialdehyde; MF, molecular functions; NAS, NAFLD activity score; NIH, National Institutes of Health; Nrf2, Nuclear factor erythroid 2-related factor 2; OB, oral bioavailability; OTUs, operational taxonomic units; p-ACC, phosphorylated acetyl-CoA carboxylase; p-AMPK, phosphorylated AMP-activated Protein Kinase; PCA, principal component analysis; PCoA, principal co-ordinates analysis; PDB, program database file; PLS-DA, Partial Least Squares Discriminant Analysis; PPI, protein–protein interaction; PVDF, polyvinylidene fluoride; qPCR, quantitative real-time PCR; ROS, reactive oxygen species; *Scd1*, stearoyl-CoA desaturase 1; SDS-PAGE, sodium dodecyl sulfate-polyacrylamide gel electrophoresis; SOD, superoxide dismutase; *Srebp1*, sterol regulatory element binding protein 1; TC, total cholesterol; TCM, Traditional Chinese Medicine; TCMSP, Traditional Chinese Medicines Systems Pharmacology Database and Analysis Platform; TG, triglyceride; TNF-α, tumor necrosis factor-alpha; XCHT, Xiaochaihutang.
