# Supplementary material for: Xiaochaihutang Extract Ameliorates Metabolic Dysfunction-Associated Steatohepatitis in Mice and Modulates the Gut–Liver Axis
Source: Antioxidants (Basel). 2026 Oct 1;15(10):1268. doi: 10.3390/antiox15101268 (PMC13700607; doi:10.3390/antiox15101268)
Supplement: Supplementary file 1 [file antioxidants-15-01268-s001.zip › antioxidants-4577542-supplementary.pdf]

Xiaochaihutang Extract Ameliorates Metabolic Dysfunction-Associated Steatohepatitis in Mice and Modulates the Gut–Liver Axis

Jiajia Yu, Zhiyuan Liu, Yanping Li, Zhen Liu, Cen Xiang * and Yuou Teng *

School of Bioengineering, Tianjin University of Science and Technology, Number 9, 13Th Street, Tianjin Economic-Technology Development Area, Tianjin 300457, China; 15122170121@163.com (J.Y.); zhiyuanliu2023@163.com (Z.L.); 15295862096@163.com (Y.L.); liuzhen5957@tust.edu.cn (Z.L.)

*Correspondence: 17909017@mail.tust.edu.cn (C.X.); tyo201485@tust.edu.cn (Y.T.); Tel.: +86-22-60912562 (C.X.); Fax: +86-22-60602298 (Y.T.)

Content

Table S1. The primer sequence of target gene.. 2

Table S2 Topological properties of the top 10 active ingredients... 3

Table S3 Information of the positive ion compounds from XCHT tentatively characterized by UHPLC-QTOF-MS 4

Table S1. The primer sequence of target gene

Gene	Primer forward (5′−3′)	Primer reverse (5′−3′)	NCBI RefSeq Accession
Tnf-α	CTGAACTTCGGGGTGATCGG	GGCTTGTCACTCGAATTTTGAGA	NM_001278601.1
Il-6	TCTATACCACTTCACAAGTCGGA	GAATTGCCATTGCACAACTCTTT	NM_001314054.1
Il-1β	CCGTGGACCTTCCAGGATGA	GGGAACGTCACACACCAGCA	NM_008361.4
Cxcl10	ATGACGGGCCAGTGAGAATG	ATGATCTCAACACGTGGGCA	NM_021274.2
Cxcl2	GCGCCCAGACAGAAGTCATA	CAGTTAGCCTTGCCTTTGTTCA	NM_009140.2
Fasn	TGGGTTCTAGCCAGCAGAGT	ACCACCAGAGACCGTTATGC	NM_007988.3
Acly	GGCTGGAGCGATCCGAAGAG	CACGGGTGGTCCTGCAGCA	NM_001199296.1
Acc	CGTGTAATGAGTAGCCAGTTAGAG	CGATCTATCCGTCGGTGGTC	NM_133360.3
Hmgcr	ATCATGTGCTGCTTCGGCTGCAT	AAATTGGACGACCCTCACGGCT	NM_001360165.1
Fabp1	TGGTCCGCAATGAGTTCACCCT	CCAGCTTGACGACTGCCTTGACTT	NM_017399.5
Srebp1	CCTGCCTTCAGGCTTCTCAGG	GAGGCCAAGCTTTGGACCTGG	NM_001313979.1
Scd1	TCTTCCTTATCATTGCCAACACCA	GCGTTGAGCACCAGAGTGTATCG	NM_009127.4
Cd36	GACTGGGACCATTGGTGATGA	AAGGCCATCTCTACCATGCC	NM_001159555.2
Cpt1α	AGGACCCTGAGGCATCTATT	ATGACCTCCTGGCATTCTCC	NM_013495.2

Table S2 Topological properties of the top 10 active ingredients

Active ingredient	MOL ID	Intermediary centrality	Proximity centrality	Degree
quercetin	MOL000098	0.3196	0.3963	281
Kaempferol	MOL000422	0.1122	0.3693	116
sitosterol	MOL000359	0.0586	0.3468	96
Stigmasterol	MOL000449	0.0577	0.3421	93
beta-sitosterol	MOL000358	0.0589	0.3417	91
Baicalein	MOL002714	0.0485	0.3241	49
Isorhamnetin	MOL000354	0.0415	0.3473	28
stepharine	MOL012921	0.0160	0.2605	25
naringenin	MOL004328	0.0629	0.3277	24
Cavidine	MOL002670	0.0251	0.2943	22

Table S3 Information of the positive ion compounds from XCHT tentatively characterized by UHPLC-QTOF-MS

No.	Compound name	Molecular formula	Expected Mass Spectra (m/z)		Measured m/z	ppm	tR (min)	MS/MS	Classification
1	5-Hydroxy-6,7,8,3′,4′-pentamethoxyflavone	C20H20O8	388.11582	-H	387.11273	10.8	1.11	179.0584、161.0485、149.0490	Flavonoid
2	5-Hydroxymethylfurfural	C6H6O3	126.03169	+H	127.03914	1.3	1.18	109.0318、69.0368	Furanoids
3	nicotinamide	C6H6N2O	122.04801	+H	123.05529	0	1.34	123.0919、80.0522	Alkaloid
4	Uracil	C4H4N2O2	112.02728	+H	113.03493	3.3	1.36	95.0225、94.0307、76.0213	Nucleoside
5	Benzoic acid	C7H6O2	122.03678	+H	123.04426	1.7	1.44	91.0565	Organic acid
6	trans-4-Hydroxycinnamic acid	C9H8O3	164.04734	+H	165.05468	0.4	1.47	165.0556、136.0769、91.0567	Coumarin
7	Adenosine	C10H13N5O4	267.09675	+H	268.1042	0.6	1.51	137.0640	Nucleosid
8	gancaonin P-3'-methyl ether	C21H20O7	384.1209	-H	383.11645	7.4	2	383.1200、341.1124、161.0505、101.0284	Flavonoid
9	Cinnamic acid	C9H8O2	148.05243	+H	149.06009	2.6	2.57	121.0300	Organic acid
10	Methyl 2-(formylamino)benzoate	C9H11NO2	165.07898	-H	164.07304	8.1	2.83	164.0745、147.0480、146.0628	Benzoate Derivatives
11	Cyclo(Ile-Ala)	C9H18N2O4	218.12666	+H	219.13396	0.1	3.02	219.1755、203.1432、86.0983、60.0492	Peptide
12	Vanillic acid	C8H8O4	168.04226	-H	167.03606	6.4	3.16	167.0408、152.0149、123.0498、108.0257	Organic acid
13	4-Hydroxy-2,5-diMethoxybenzaldehyde	C9H10O4	182.05791	-H	181.05147	4.6	3.28	181.0498、137.0631、135.0480、121.0317	Phenolic aldehydes
14	Syringic acid	C9H10O5	198.05282	+H	199.0599	-1	3.4	198.0936、198.0766、170.0812、70.0696	Organic acid

15	Acetovanillone	C9H10O3	166.06299	-H	165.05744	10.4	4.36	165.0579、93.0397	Acetophenone derivatives
16	Chlorogenic acid	C16H18O9	354.09508	+H	355.10273	1	4.7	145.0287	Organic acid
17	Epicatechin	C15H14O6	290.07904	+H	291.08654	0.8	4.7	147.0445	Flavonoid
18	7-Hydroxycoumarin	C9H6O3	162.03169	+H	163.03905	0.5	4.7	163.0391、145.0293	Coumarin
19	Salicylic acid	C7H6O3	138.03169	+H	139.03906	0.7	4.7	139.0393	Organic acid
20	Catechin	C15H14O6	290.07904	-H	289.07176	0	4.82	289.0733、245.0845、203.0734、123.0488、109.0336	Flavonoid
21	Isoquercetin	C21H20O12	464.09548	+H	465.10317	0.9	5.28	303.0513	Flavonoid
22	Quercetin-7-O-β-D-glucopyranoside	C21H20O12	464.09548	-H	463.08596	-4.8	5.34	463.0891、229.0514	Glycosid
23	Vanillic acid	C8H8O4	168.04226	+H	169.04955	0.1	5.34	169.0751、151.0374、142.0667	Organic acid
24	Chlorogenic acid	C18H24O12	432.12678	+H	433.13442	0.9	5.46	145.0506	Organic acid
25	Coclaurine	C17H19NO3	285.13649	+H	286.14363	-0.5	5.67	175.0760、254.0934、286.1459	Alkaloid
26	Luteolin 7-glucoside	C27H30O15	594.15847	+H	595.16566	-0.1	5.92	457.1129、349.07389、335.0933、307.0580	Glycosid
27	6-O-methylcatalpol	C16H24O10	376.13695	+H	377.14571	3.9	5.93	377.1453、359.1423	Iridoid glycosides
28	N-Methyl-ephedrine	C11H16O5	228.09977	+H	229.10719	0.6	5.98	229.0973、133.0650、81.0725	Alkaloid
29	N-Methylcamphorine	C20H23NO4	341.16271	+H	342.17033	1	6.11	342.1713、297.1129、265.0863	Alkaloid
30	(6S,9S)-Longiflorigenin	C19H30O8	386.19407	+H	387.20172	1	6.14	387.2109、369.1187、175.0389、95.0873	Glycosid

31	Glycyrrhizin G	C21H20O5	352.13107	-H	351.12835	13	6.32	351.1302、249.0631	Flavonoid
32	Dihydroquercetin	C15H12O7	304.0583	-H	303.05104	0	6.44	303.0526、177.0217	Flavonoid
33	8-Methoxypsoralen	C12H8O4	216.04226	+H	217.04946	-0.4	6.6	217.0493、189.0513	Coumarin
34	Vitexin-1	C26H28O14	564.14791	+H	565.1557	0.9	6.66	565.1553、409.0934、391.0839、321.0774、295.0607	Flavonoid
35	Vanillin	C8H8O3	152.04734	+H	153.05476	0.9	6.77	153.0923、83.0878	Phenolic aldehydes
36	Quercetin 3-O-glucoside	C21H18O13	478.07474	-H	477.06559	-3.9	6.8	301.0366	Glycosid
37	Naringin	C27H32O14	580.17921	-H	579.16891	-5.2	6.93	579.1724、255.0681	Flavonoid
38	Heptacoside	C9H6O4	178.02661	+H	179.03396	0.4	7.07	179.0348、147.0410	Coumarin
39	Naringin	C27H32O14	580.17921	+H	581.18557	-1.6	7.28	581.1815、419.1348、257.0816	Flavonoid
40	Poncirin 4'-rhamnoside	C27H30O14	578.16356	+H	579.17137	0.9	7.47	423.1092、379.0840、325.0721	Glycosid
41	Isoscopoletin	C10H8O4	192.04226	+H	193.04947	-0.4	7.49	193.0503、178.0276	Coumarin
42	Rutin	C27H30O16	610.15339	+H	611.16122	0.9	7.5	465.1031、303.0497	lavonoid
43	4,8-Dihydroxy-1-tetralone	C10H8O3	176.04734	+H	177.0547	0.4	7.65	177.0534、149.0594、117.0351、89.0410	Naphthoquinon
44	Liquiritin apioside	C26H30O13	550.16864	-H	549.15864	-5	7.76	393.0977、363.0861	Flavonoid
45	Liquiritin	C21H22O9	418.12638	-H	417.11799	-2.7	7.83	417.1194、255.0676、135.0130、119.0552	Flavonoid
46	Quercetin 3-O-glucoside	C21H18O13	478.07474	+H	479.08156	-0.9	7.85	303.0500、285.0397、257.0450、241.0490	Glycoside
47	Hovetrichoside C	C21H22O11	450.11621	+H	451.12351	0	8.13	305.0659、259.0600、71.0525、	Glycoside
48	Dihydroquercetin	C15H12O7	304.0583	+H	305.06568	0.3	8.14	231.0658、153.0186、	Flavonoid

								149.0237	
49	Puerarin Apioside	C26H28O13	548.15299	+H	549.16008	-0.3	8.3	333.0763、321.0765、305.0820、291.0659、	Glycoside
50	20-O-Glucoginsenoside Rf	C48H82O19	962.54503	+H	963.553	0.7	8.58	421.3439、309.1202、219.1744、143.1066	Saponin
51	Verbascoside	C29H36O15	624.20542	+H	625.21293	0.4	8.64	181.0504、163.0388、	Glycoside
52	4'-Methoxyglucopyranoside	C23H24O12	492.12678	+H	493.13422	0.3	8.79	331.0818	Glycoside
53	Narcisin	C28H32O16	624.16904	-H	623.16119	-0.9	8.79	623.1986、295.0629	Flavonoid
54	Puerarin	C21H20O9	416.11073	+H	417.11803	0	9.08	417.1180、268.0744、267.0659	Flavonoid
55	Diosmin	C28H32O15	608.17412	+H	609.18074	-1.1	9.14	447.1273、285.0759、271.0612	Flavonoid
56	Hesperitin	C16H14O6	302.07904	+H	303.08629	-0.1	9.17	285.0761、137.0242	Flavonoid
57	Anhydroicaritin-7-O-glucoside	C18H26O9	386.15768	+H	387.16595	2.6	9.19	180.1137、138.0924	Flavonoid
58	2-ethoxyJuglone	C12H10O4	218.05791	+H	219.06531	0.6	9.26	203.1422、173.0602	Naphthoquinone
59	Scrophulariaceae aglycone	C9H14O3	170.09429	+H	171.10164	0.4	9.48	81.0725、72.0459	Flavonoid
60	8-Hydroxypinoresinol	C20H22O7	374.13655	+H	375.14409	0.7	9.54	137.0605	Lignans
61	Dihydrobaicalin	C16H14O6	302.07904	-H	301.07182	0.2	9.73	161.0260	Flavonoid
62	Cistanoside C	C30H38O15	638.22107	+H	639.22739	-1.5	10	639.1579、339.1121、177.0548	Triterpenoid glycosides
63	2''-Acetylhyperin	C23H22O13	506.10604	+H	507.11321	-0.2	10.32	331.0817、316.0594、	Alkaloid
64	6''-Acetylhyperin	C23H22O13	506.10604	+H	507.11321	-0.2	10.32	301.0350	Alkaloid
65	Baicalin	C21H18O11	446.08491	+H	447.09168	-1.1	10.48	271.0597	Flavonoid
66	Ononin	C22H22O9	430.12638	+H	431.13366	0	10.57	269.0808	Flavonoid
67	Apigenin 7-glucoside	C21H20O10	432.10565	+H	433.11278	-0.3	10.58	270.0846、269.0814	Glycoside
68	ginsenoside RH 4	C36H60O8	620.42882	+H	621.43655	0.7	10.62	203.1794、129.0545	Saponin

69	Licorice glycoside D2	C35H36O15	696.20542	-H	695.19587	-3.3	10.71	531.1517、255.0684	Glycoside
70	Licorice glycoside C1	C36H38O16	726.21599	-H	725.20539	-4.6	10.79	255.0679	Glycoside
71	Tetrahydro-2-(4-hydroxy-3-methoxyphenyl)-4-((4-hydroxy-3-methoxyphenyl)methyl)-3-furanemethanol	C20H24O6	360.15729	-H	359.1499	-0.3	10.89	193.0892	Lignans
72	Liquiritigenin	C15H12O4	256.07356	-H	255.06654	1	10.94	91.0262	Flavonoid
73	Luteolin 7-O-β-D-glucoside	C21H20O11	448.10056	-H	447.09161	-3.8	11.11	448.0978、271.0640、175.0279、113.0308	Anthraquinone
74	1-Hydroxy-2-methylanthraquinone	C15H10O3	238.06299	+H	239.07049	0.9	11.17	239.0717、165.0713	Anthraquinone
75	Baicalin	C21H18O11	446.08491	-H	445.07635	-2.9	11.33	269.0466	Flavonoid
76	Quercetin	C15H10O7	302.04265	+H	303.05022	1	11.38	303.0506、229.0505、153.0195	Flavonoid
77	Cistanoside D	C31H40O15	652.23672	+H	653.24427	0.4	11.39	653.1371、339.1106、301.0717、177.0542	Terpenoids
78	Torachrysone 8-O-glucoside	C21H20O10	432.10565	-H	431.09757	-1.9	11.61	431.1002、255.0676、152.9992	Glycoside
79	Chrysin 7-O-β-galactopyranuronoside	C21H18O10	430.09	+H	431.09722	-0.1	11.81	431.0973、255.0648	Glycoside
80	Ononin	C22H22O9	430.12638	-H	429.08171	-87.1	11.82	253.0531、113.0297	Flavonoid
81	Dicaffeoylquinic acid	C25H24O12	516.12678	+H	517.13391	-0.3	11.88	517.1372、269.0815	Phenolic acid
82	5,7,8, 3′,4′-Pentamethylflavone	C20H20O7	372.1209	-H	371.11348	-0.4	12.14	371.1154、303.1262	Flavonoid
83	Physcion 8-β-D-glucoside	C22H22O11	462.11621	+H	463.12128	-4.8	12.28	462.1109、285.0753	Saponin
84	Gingerenone A	C21H24O5	356.16237	+H	357.16986	0.6	12.49	177.0922、137.0610	Phenols
85	Licoricesaponin A3	C48H72O21	984.45661	-H	983.44521	-4.2	12.62	983.4495、821.3980	Saponin
86	5,7,4’-trihydroxyflavanone	C15H12O5	272.06847	+H	273.07591	0.6	12.67	153.0179、147.0440	Flavonoid

87	(2E)-3-(4-hydroxy-2-methoxyphenyl)-1-(4-hydroxyphenyl)prop-2-en-1-one	C16H14O4	270.08921	-H	269.0822	1	12.67	269.0468、181.0676、133.0328	Chalcones
88	β-D-Glucopyranoside	C48H78O18	942.51882	+H	943.5258	-0.3	12.83	599.3956、583.3968、455.3518、437.3417、419.3319、273.0984、147.0662	Glycoside
89	γ-Terpinene	C10H16	136.1252	+H	137.1326	0.9	12.85	94.0433、66.0493	Terpenoid
90	Kaempferol	C15H10O6	286.04774	+H	287.05521	0.7	12.86	287.0548、213.0550	Flavonoid
91	Anthocyanidin	C15H11O6	287.05556	+H	288.05922	-12.6	12.86	287.0548、153.0185	Flavonoid
92	5,6-Dihydroxy-7,3,4-trimethoxyflavone	C18H16O8	360.08452	+H	361.09214	0.9	12.86	346.0701、197.0091、169.0141	Flavonoid
93	Oleanolic acid	C30H48O3	456.36035	+H	457.36785	0.5	13.06	457.1828、421.3486、143.1072	Terpenoids
94	Ginsenoside Rg1	C42H72O14	800.49221	+H	801.49908	-0.5	13.07	783.4914、143.1076、125.0973	Saponin
95	Isorhamnetin	C16H12O7	316.0583	+H	317.06582	0.7	13.07	317.0661、153.0189	Flavonoid
96	Ginsenoside-Rg6	C42H70O12	766.48673	+H	767.49333	-0.9	13.08	767.4956、217.1955、207.1746	Saponin
97	Ginsenoside-RH3	C36H60O7	604.4339	+H	605.44116	0	13.08	217.1955、207.1746	Saponin
98	Ginsenoside-Rg1	C42H72O14	800.49221	-H	799.48219	-3.4	13.09	269.0460	Saponin
99	Ginsenoside-Rb3	C53H90O22	1078.59238	-H	1077.58175	-3.1	13.25	962.2214	Saponin
100	Fallacinol	C16H12O6	300.06339	-H	299.05629	0.6	13.32	182.6863、167.6626	Terpenoids
101	Ginsenoside-RO	C48H76O19	956.49808	-H	955.48724	-3.7	13.43	839.1192	Saponin
102	Baicalein	C15H10O5	270.05282	-H	269.04628	2.7	13.46	270.0503、269.0469、251.0372	Flavonoid
103	Licorice saponin G2	C42H62O17	838.3987	-H	837.38924	-2.6	13.6	722.0239	Saponin

104	Saikosaponin I	C48H78O17	926.5239	+H	927.5308	-0.4	13.61	927.4687、273.1102、147.0646	Saponin
105	Cis-Carvylacetate	C12H18O2	194.13068	+H	195.1378	-0.8	13.64	105.0723、93.0720、91.0555、77.0418	Monoterpenes
106	9(S),10(S),11(R)-trihydroxy-12(Z)-octadecenoic acid	C18H34O5	330.24062	-H	329.23327	-0.2	13.65	212.8639	Organic acid
107	Pseudoginsenoside RT1	C47H74O18	926.48752	-H	925.47934	-1	13.68	809.1156、453.0207	Saponin
108	Formononetin	C16H12O4	268.07356	-H	267.06663	1.3	13.88	135.6740	Flavonoid
109	Ginsenoside Rd	C48H82O18	946.55012	-H	945.53985	-3.2	13.88	829.1736	Saponin
110	Tectochrysin	C16H12O4	268.07356	+H	269.08109	0.9	13.89	225.0557、213.0924、181.0662	Flavonoid
111	Chikusetsu saponin IVA	C42H66O14	794.44526	-H	793.43447	-4.4	13.93	678.0604	Saponin
112	7,3'-Dihydroxy-4'-Methoxyflavan	C16H16O4	272.10486	-H	271.09784	1	13.93	154.7274	Flavonoid
113	(2α,3β)-2,3-Dihydroxy-11,13(18)-dien-28-oic acid	C30H46O4	470.33961	+H	471.34644	-0.9	14.02	471.3468、317.2135、235.1697	Triterpenoids
114	Glycyrrhizic acid	C42H62O16	822.40379	-H	821.39389	-3.2	14.04	823.4079、471.3467、453.3356、407.3318、317.2135	Saponin
115	Licorice-saponin H2	C42H62O16	822.40379	-H	821.39389	-3.2	14.04	821.3954、351.0573	Saponin
116	Ginsenoside-RO	C48H76O19	956.49808	+H	957.50461	-0.8	14.35	409.3449、397.3136、309.1195、159.0305	Saponin
117	Licorice-saponin B2	C42H64O15	808.42452	-H	807.41493	-2.9	14.4	691.0446	Saponin
118	Uridine	C15H24	204.1878	+H	205.19495	-0.6	14.47	205.0722	Nucleoside
119	Caryophyllene	C15H24	204.1878	+H	205.19495	-0.6	14.47		Terpenoids
120	Saikosaponin D	C42H68O13	780.46599	-H	779.45687	-2.4	14.64	779.4574、617.4071	Saponin
121	Saikosaponin B2	C42H68O13	780.46599	+H	781.47246	-1	14.65	455.3516	Saponin

122	Prosaikogenin A	C36H58O8	618.41317	+H	619.42038	-0.1	14.65	455.3539、437.3429、397.3128、267.2141、177.1635	Saponin
123	Wogonin	C16H12O5	284.06847	-H	283.06144	0.9	14.76	283.0621、268.0388	Flavonoid
124	1-Methyl Emodin	C16H12O5	284.06847	+H	285.07586	0.4	14.77	285.0762、284.0322、270.0525、179.0497	Flavonoid
125	Chrysophanic acid	C15H10O4	254.05791	-H	253.05085	0.9	14.86	137.6849、27.6843	Anthraquinone
126	Rubiadin	C15H10O4	254.05791	+H	255.06508	-0.4	14.86	255.0650、153.0184	Anthraquinone
127	Salvianolic acid F	C17H14O6	314.07904	+H	315.08654	0.7	14.89	285.0393、257.0454	Organic acid
128	Skullcapflavone II	C19H18O8	374.10017	-H	373.09226	-1.7	14.98	373.0930、358.0697、343.0467、194.9963	Flavonoid
129	Licoricesaponin F3	C48H72O19	952.46678	-H	951.45613	-3.5	14.99	835.0909	Saponin
130	Bavacoumestan A	C20H16O6	352.09469	+H	353.1022	0.7	15.01	311.0556、299.0559	Coumestans
131	Kumatakenin	C17H14O6	314.07904	-H	313.07177	0	15.05	197.7063	Flavonoid
132	6-Shogaol	C17H24O3	276.17254	+H	277.17992	0.4	15.06	177.0914、145.0654	Alkylphenol
133	4'-Hydroxyacetophenone	C8H8O2	136.05243	+H	137.06017	3.4	15.06	94.0439	Phenolic ketones
134	6-Gingerol	C17H26O4	294.18311	+H	295.19044	0.2	15.06	177.0911、137.0600	Phenolic
135	Bupleurotoxin	C17H22O2	258.16198	+H	259.16927	0	15.07	259.1702	Saponin
136	Licoflavonol	C20H18O6	354.11034	-H	353.10244	-1.8	15.13	236.7327	Flavonoid
137	2-(4-Hydroxy-3-methoxybenzoyl)-4-methoxybenzaldehyde	C16H14O5	286.08412	-H	285.07681	-0.1	15.17	169.7081	Phenolic aldehydes
138	Glycycoumarin	C21H20O6	368.12599	-H	367.11793	-2.1	15.25	180.6703	Coumarin
139	Licorice glycoside C2	C42H62O15	806.40887	-H	805.39881	-3.5	15.26	689.0321	Flavonoid

140	Scutellarin	C18H16O7	344.0896	-H	343.0819	-1.3	15.35	153.6481	Flavonoid
141	poricoic acid B	C30H44O5	484.31887	-H	483.30974	-3.8	15.44	366.9414、184.8476	Organic acid
142	Goshonoside	C30H44O5	484.31887	+H	485.32568	-1	15.45	485.3255、421.3093	Flavonoid
143	6"-O-acetylsaikosaponin D	C44H70O14	822.47656	+H	823.48207	-2.1	15.57	823.4836、453.3372、437.3415	Saponin
144	farnesal	C15H24O	220.18272	+H	221.18996	-0.1	15.61	221.1911、203.1785、193.1619、133.1024、119.0873、105.0730	Terpenoids
145	Bavachalcone	C21H22O4	338.15181	+H	339.15907	-0.1	15.83	339.1605、297.1507	Flavonoid
146	Licochalcone-A	C21H22O4	338.15181	-H	337.14421	-0.9	15.83	220.7748、188.7482、112.7183、70.7074	Flavonoid
147	Ginsenoside RH4	C36H60O8	620.42882	-H	619.41933	-3.6	15.88	503.0511、138.8658、36.6265	Saponin
148	20(R)-Ginsenoside Rg3	C42H72O13	784.49729	-H	783.48685	-4	15.93	667.1207、505.0726	Saponin
149	Glycyrol	C21H18O6	366.11034	-H	365.10276	-0.8	15.98	186.8202	Flavonoid
150	3”，6”—O,O-Diacetylsaikosaponin b2	C46H72O15	864.48712	+H	865.49453	0.1	16.19	865.4855、583.4074、455.3536、437.3427、247.0840、187.0625	Saponin
151	Desaminotyrosine	C9H10O3	166.06299	+H	167.0704	0.8	16.37	121.0294、152.0649	Organic acid
152	Poricoic acid G	C30H46O5	486.33453	-H	485.32554	-3.5	16.38	367.9427、305.9474、138.8635、58.6815	Organic acid
153	Glabrone	C20H16O5	336.09977	-H	335.09237	-0.4	16.4	218.7224、174.7315、82.7062	Flavonoid
154	Isopsoralidin	C20H16O5	336.09977	+H	337.10709	0.1	16.4	337.1073、239.0703	Coumarin
155	Corylin	C20H20O4	324.13616	+H	325.14353	0.3	16.52	325.1403、189.0905、187.1201	Flavonoid

156	Semilicoisoflavone B	C20H16O6	352.09469	-H	351.08741	0	16.75	234.7170、166.7278、124.7175、82.7073	Flavonoid
157	Saikosaponin A	C30H48O4	472.35526	+H	473.36244	-0.2	17.17	473.3636、437.3444、383.2952、365.2838	Saponin
158	Saikosaponin D	C30H48O4	472.35526	-H	471.3461	-4	17.52	354.9775	Saponin
159	Saikosaponin B3	C43H72O14	812.49221	-H	811.48179	-3.9	17.6	695.1152、649.1105	Saponin
160	Ginsenoside RG5	C42H70O12	766.48673	-H	765.47955	0.1	17.6	650.1152、488.0570、138.8685、44.6768	Saponin
161	6″-O-Acetyl Saikosaponin B3	C45H74O15	854.50277	-H	853.49297	-3	17.61	529.0582	Saponin
162	Icariin B	C25H26O6	422.17294	+H	423.17985	-0.9	17.83	423.1801、253.0659、124.0647	Flavonoid
163	6-Gingerdione	C17H24O4	292.16746	+H	293.17496	0.8	18.23	293.1775、237.1117、181.0505	Flavonoid
164	Glycyrrhetinic acid	C30H46O4	470.33961	-H	469.33031	-4.3	18.4	353.9644、309.9766	Saponin
165	Germacrene D	C15H26O	222.19837	+H	223.20582	0.8	19.65	223.2085、207.0325、189.0199、73.0514	Terpene
166	beta-Sitosterol	C29H50O	414.38617	+H	415.3896	-9.2	19.84	415.3576、319.3021	sterol
167	16-Deoxysaikogenin F	C30H48O3	456.36035	-H	455.35115	-4.2	19.85	339.9856、267.9886、254.9904	Terpenoids
168	alpha-Spinastero	C29H48O	412.37052	+H	413.37755	-0.6	20.12	413.3797、395.3678	sterol
169	Miltirone	C19H22O2	282.16198	+H	283.17502	20.4	21.44	283.0457	Flavonoid
170	Methyl Pentadecanoat	C16H32O2	256.24023	-H	255.23312	0.6	21.83	138.8630	Fatty acid ester
171	Pseudoginsenoside Rh2	C36H62O8	622.44447	-H	621.43512	-3.3	23.94	504.9935	Saponin
